# Digital psychiatry in Nigeria: A scoping review

**DOI:** 10.4102/sajpsychiatry.v30i0.2115

**Published:** 2024-03-21

**Authors:** Justus U. Onu, Tonia C. Onyeka

**Affiliations:** 1 Department of Mental Health, Faculty of Medicine, Nnamdi Azikiwe University, Awka, Nigeria; 2 Center for Translation and Implementation Research (CTAIR), Faculty of Medical Sciences, University of Nigeria, Enugu, Nigeria; 3 Department of Anaesthesia, Pain and Palliative Care Unit, Faculty of Medical Sciences, University of Nigeria, Enugu, Nigeria

**Keywords:** digital psychiatry, digital healthcare, telemedicine, mental health services, Nigeria

## Abstract

**Background:**

Mental healthcare workforce shortage in Nigeria poses a major obstacle to mental health services scale-up. Digital psychiatry may provide a veritable platform to bridge treatment gaps.

**Aim:**

To provide an overview of quantity and range of peer-reviewed publications on digital psychiatry in Nigeria.

**Setting:**

A comprehensive literature search encompassed all original, peer-reviewed research articles on digital psychiatry in Nigeria. PubMed, Google Scholar, and a direct exploration of relevant journal article reference lists were utilised. Inclusion criteria covered peer-reviewed original articles conducted in Nigeria between January 2013 and January 2023, regardless of quality. Exclusions comprised case reports, reviews, dissertations, and abstracts.

**Methods:**

Preferred Reporting Items for Systematic Reviews and Meta-Analyses Extension for Scoping Reviews (PRISMA-ScR) guidelines were adhered to, while methodological framework of Arksey and O’Malley was used to describe the review.

**Results:**

Fourteen studies meeting inclusion criteria exhibited two primary research areas: implementation and intervention. Most studies focused on intervention strategies, showcasing efficacy of digital devices in enhancing outcomes in depression and clinic appointments. Implementation studies indicated favorable acceptance by both clients and healthcare practitioners.

**Conclusion:**

Digital technology seems acceptable to Nigerian patients and clinicians. Policies to operationalise provision of digital healthcare services will have positive impact in addressing unmet mental health needs. Finally, the quality of the evidence from majority of studies has to be enhanced, and additional studies are required to uncover gaps in some regions of the country.

**Contribution:**

This research demonstrates that, despite some drawbacks, digital methods of providing mental healthcare are practical in Nigeria.

## Introduction

In sub-Saharan Africa (SSA), mental, neurological and substance (MNS) use disorders account for over 350 million disability-adjusted life years (DALYs) lost each year, which is more than the 150 million DALYs experienced in industrialised nations.^1^ One in four Nigerians, or over 60 million people, is known to experience some form of mental illness at some point in their lives.^2^ According to estimates made by Charlson et al.,^3^ the burden of MNS in SSA countries will likely increase by 130% by the year 2050 as a result of epidemiologic and demographic changes. In spite of these ominous forecasts, the treatment gap continues to be significant, with over 75% of individuals residing in low- and middle-income countries, like Nigeria.^4,5^ One epidemiological survey involving multiple countries, which included Nigeria, revealed that a mere 20% of individuals suffering from common mental illnesses had sought treatment within the previous year.^5^ The bulk of the Nigerian population sought for care in religious and traditional settings, with only 10% obtaining the barest minimum mental health treatment.^5^ This treatment gap is caused by a number of factors, one of which being Nigeria’s sparse mental health workforce.^5^ With a population of over 200 million, Nigeria has less than 300 psychiatrists, translating to a psychiatrist to population ratio of over 700 000.^6^ For other mental healthcare specialists like clinical psychologists, psychiatric nurses and occupational therapists, the situation is far worse.^6^ The unequal migration both within and outside of Nigeria poses a further danger to the country’s mental health workforce with overconcentration of specialists in the urban cities.^6^ Thus, the vast majority of communities in Nigeria are left without any form of specialised mental health services.

Major challenges to providing appropriate mental healthcare and expanding services to overcome treatment gaps in Nigeria are posed by labour shortages.^5^ Digital technologies have been considered to offer an alternative or complement the traditional methods of mental healthcare delivery.^7^ In this review, we define digital psychiatry as any application of digital health technology for mental health assessment, support, prevention and treatment.^8^ It can be either synchronous (i.e. a live, remote exchange of patient data involving direct, in-person communication between a doctor and a patient) or asynchronous (i.e. a ‘store-and-forward’ strategy in which the patient shares data via a patient portal and the doctor reviews it later).^9^ According to predictions by Tse et al.^6^ and Cosh et al.,^7^ digital psychiatry will be a real platform for enhancing mental health services through supervision, collaborative consultation, bettering medication adherence and clinic attendance, supporting diagnosis and providing therapy. There may be new chances to take advantage of these digital resources to enhance the delivery of mental healthcare in Nigeria as a result of the country’s rapidly growing access to and use of digital technologies. The Global System for Mobile Communication Association (GSMA) recently released a report estimating that in 2018, there were over 747 million mobile phone subscribers and over 302 million smartphone users.^10^ This number is expected to increase to over 1 billion by 2025.^10^ For those with mental, neurological and drug use disorders, this offers a variety of options for low- to medium-level therapies. There are some data to support the use of mobile technology in outpatient clinic treatment, psychotherapy and relapse prevention in Nigeria.^11,12,13^

Despite this acknowledgement of robust digital penetration in Nigeria and the limited number of studies that have looked at the use of digital technologies in mental health services in Nigeria, there is a dearth of an integrative synthesis of the body of literature. The authors think that a scoping review is an ideal first step towards identifying existing gaps in the literature and that the scoping review process helps in refining the inclusion and exclusion criteria for the systematic review. By conducting a scoping review, researchers can assess the feasibility of conducting a systematic review, in relation to sufficient volume of literature on the topic, whether the available evidence is diverse enough, and whether the research question is answerable through a systematic review. In addition, findings from a scoping review can inform the development of the systematic review protocol.^14^ Therefore, understanding the quantity of research and the subject areas with sufficient studies to support a systematic review and meta-analysis in order to develop the evidence for upcoming policy recommendations is necessary. This is the first scoping review of the subject in Nigeria, as far as the authors’ knowledge goes. This scoping review sought to address the question: What is the extent and quantity of research on digital psychiatry in Nigeria that has been published in peer-reviewed journals?

## Information sources, search strategy and selection criteria

**FIGURE 1:**
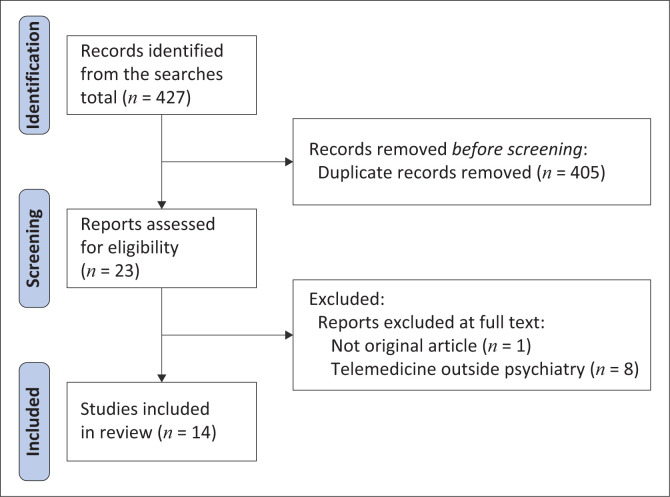
Articles selection process.

In accordance with the Preferred Reporting Items for Systematic Reviews and Meta-Analyses Extension for Scoping Reviews (PRISMA-ScR) guidelines,^15^ study results were reported to ensure the transparent and comprehensive reporting of this scoping review. Also, the PRISMA-ScR checklist was systematically followed to guide the planning, execution and reporting of our scoping review methodology. Scoping reviews, according to Sargeant et al.,^16^ are usually descriptive reviews, designed to chart the literature around a particular topic. The approach involves an extensive literature search, followed by a structured mapping, or charting, of the literature. The results of scoping reviews not only help to inform future research by identifying gaps in the existing literature but also are used to identify areas where there may be a sufficient depth of literature to warrant a systematic review.^16^ This review aimed to establish the quantity and breadth of peer-reviewed original research articles on digital psychiatry in Nigeria, in order to articulate evidence-based recommendations for future research and clinical priority setting in the country. The review used the methodological framework outlined by Arksey and O’Malley,^17^ which entails determining the research question (step 1), locating relevant studies (step 2), choosing relevant studies (step 3), organising and charting data (step 4), and summarising and communicating the findings (steps 5 and 6). The following steps were followed:

- Step 1 – Identifying the research question: For the purpose of this scoping review, an all-embracing research question was chosen to ensure that all pertinent publications were included. The question was related to the aim of the review, which is to describe the amount of research and the breadth of published peer-reviewed articles on digital psychiatry in Nigeria.
- Step 2 – Identifying relevant studies: Each author independently performed searches in PubMed for all peer-reviewed original research articles published in the past decade relating to digital psychiatry in Nigeria which met the eligibility criteria. The inclusion criteria covered peer-reviewed original articles conducted in Nigeria from January 2013 to January 2023, regardless of their quality. Excluded were case reports, reviews, dissertations and abstracts. Searches were also conducted on Google Scholar, and direct searches of reference lists of pertinent journal articles were also carried out. Thereafter, a joint search was conducted to ensure rigour. Specific words and phrases like digital psychiatry, telemental health, telemedicine, telepsychiatry, short message service (SMS), text messages, voice messages, smartphones, phone calls, mental health apps, video conference, training, intervention, treatment, diagnosis, prevention, follow-up, missed appointment, adherence, mental health, mental healthcare services, suicide, depression, anxiety, psychosis and Nigeria were combined with Boolean operators for the searches in Google Scholar. In PubMed, the building block approach (i.e. the three-step process) was used to conduct the search: firstly, each search term and phrase was sought for individually using the MeSH subject heading; secondly, the MeSH terms and keywords were added to the history; and lastly, a chart builder was used to combine the histories created (Table 1).
- Step 3 – Study selection: Because of the need to quantify the volume and breadth of the literature, articles were not excluded on the basis of quality. However, case-based reports and review articles were excluded. Citations were imported into EndNote and 405 duplicate articles were filtered out. Relevant publications identified after removing duplicates were screened for other eligibility criteria (Figure 1). The authors (J.U.O. and T.C.O.) independently reviewed each title and abstract for inclusion. When a decision was not possible at this point, the entire article was read and a decision was made. The writers convened jointly and reached a consensus on the final papers to be included in this review. All publications that met the inclusion criteria had their full texts reviewed, and the selection process is shown in Figure 1.
- Step 4 – Charting the data: After assembling and reading through full texts of all eligible articles, the authors (J.U.O. and T.C.O.) agreed on two major domains of fit, namely, implementation and interventions. Extracted data (first author name, year of publication, study characteristics, summary of main findings and area of focus) from each of the article was entered in Microsoft Excel data collection sheet.
- Step 5 – Synthesis of findings: After carefully reading an article, each author used one phrase – a code – to distil the main conclusions. A number of virtual platform-based meetings were used to reconcile the summary of findings that each author had independently made through a consensus.

**TABLE 1:** Search strategy.

Category	Search parameters
Population	“Digital psychiatry*”[tw] OR “psychiatry”[tw] OR e-psychiatry”[tw] OR “mobile psychiatry*”[tw] OR e-psychiatry”[tw] OR “mobile psychiatry*”[tw]
Intervention	Text Messaging*”[MeSH] OR “sms”[tw] OR “short message service*” OR “Videoconferencing”[MeSH] OR “Teleconferencing”[tw] OR “audio conferencing*”[tw] OR “remote supervision*”[tw] OR “Artificial intelligence*”[MeSH] OR “Digital technology*”[MeSH:NoExp] OR “smart-phone based*”[tw] OR “mobile technology”[tw] OR “mHealth”[tw] OR “mobile healthcare*”[tw] OR “digital technology*”[tw] OR “digital technologies*”[tw] OR “digital health*”[tw] OR “mobile health intervention*”[tw] OR “mobile health technologies*”[tw] OR “artificial intelligence*”[tw] OR “phone calls*”[tw] OR “smartphone”[MeSH] OR “Mobile applications*”[MeSH] OR “smartphones”[tw] OR “Cell phone”[MeSH] OR “Cell phone use*”[MeSH] OR Mobile phone*”[tw] OR “mobile medical application*”[tw] OR “mobile app*”[tw] OR “mobile apps*”[tw] AND “Telemedicine”[MeSH] OR “Telemedicine”[tw] OR “Telepsychology”[tw] AND “Mental health*”[MeSH] OR “Mental health services*”[MeSH] OR “Internet-based intervention*”[MeSH] OR “Mental health intervention*”[tw] OR “internet-based intervention”[tw]
Outcome	OR “mental illness*” [tw] OR “suicide”[tw] OR “depression”[tw] OR “psychosis”[tw] AND “effectiveness”[tw] OR “efficacy”[tw] AND “Nigeria”[MeSh] OR “Nigeria”[tw] OR “West Africa”[tw] OR “LMIC”[tw] OR “sub-Saharan Africa*”[tw] AND “follow-up”[tw] AND “Medication adherence*”[MeSH] OR “feasibility”[tw] AND “adoption”[tw] AND “treatment”[MeSH] AND “diagnosis’[MeSH] AND mental Health*”[MeSH] OR “mental health services*”[MeSH]

### Ethical considerations

This article followed all ethical standards for research without direct contact with human or animal subjects. The scoping review protocol was submitted to the Ethics Committee, and because the approach primarily consisted of a literature review rather than the use of human subjects, ethical clearance was waived.

## Results

A total of 427 relevant titles were retrieved at the initial search. Following removal of duplicates, the search was further refined to produce 23 publications which were screened against the inclusion criteria. Finally, 14 publications (randomised controlled trials [*n* = 6], cross-sectional studies [*n* = 6] and qualitative design [*n* = 2]) meeting the eligibility criteria were selected. Table 2 contains the description of these studies.

**TABLE 2:** Summary of the included articles on digital psychiatry in Nigeria.

s/no	Author and year	Study characteristics	Main findings	Area of focus	Type of digital psychiatry
1.	Gureje et al., 2022	This was a two-arm parallel, cluster randomised trial involving 30 primary maternal care clinics in Ibadan, Nigeria (15 intervention arms and 15 control arms), to evaluate the effectiveness of the intervention on adolescent mothers with perinatal depression. Participants were adolescent pregnant women aged <20 years at foetal gestation of 16–36 weeks with moderate-to-severe depression. The interventions included behavioural activation, problem-solving treatment, parenting skills training, social and parenting skills support provided by a self-identified adult ‘neighborhood mother’ and *treatment adherence promoted by phone calls and sending text messages.* Primary outcomes were depression symptoms measured with EPDS and parenting practices measured with Infant-Toddler version of the Home Inventory for Measurement of the Environment (HOME-IT) assessed at 6-month postnatal period.	The intervention arm had lower level of depressive symptoms when compared with the control arm, mean EPDS score of 5.5 (s.d. −3.6) versus 7.2 (s.d. −4.0) (adjusted mean difference −1.84; 95% CI: −3.06 to −0.62; *p* = 0.003) and better parenting practices with higher score in the HOME-IT scale.	Intervention	Synchronous communications (i.e. phone calls) and asynchronous communication (i.e. text messages)
2.	Ofoegbu et al., 2020	The study determined the effectiveness of guided *internet-assisted* intervention (GIAI) on depressive symptoms among tertiary students in Nigeria. This was a group randomised trial involving 96 participants with depression per arm. It lasted for 10 weeks. Intervention arm was given GIAI which involves psychoeducation, interactive peer support, cognitive disputation, behavioural homework assignments and role play. Usual care was not specifically defined. Analysis was by repeated measures analysis of variance.	There was significant reduction in the Beck’s Depression Inventory in the intervention arm when compared to the usual care across the intervals of https://drive.google.com/drive/folders/15nbT9vyrqJPmWpdqrDoqZssPSsieZmgCfollow-up.	Intervention	Asynchronous (e.g. digital education materials) and synchronous (e.g. real-time interactive sessions)
3.	Ogbolu et al., 2020	The study described the use of *telephone* helpline in suicide prevention. A descriptive study of the first 100 calls on a 24-h suicide telephone helpline.	A hundred completed calls in the period under examination. About 47% called to report crisis situation, while 20% called to seek mental health advice. Interventions such as counselling and linkage to mental health services were given via the telephone calls.	Intervention	Synchronous (i.e. telephone conversation)
4.	Adewuya et al., 2019	Cluster randomised controlled trial. Involving 10 primary healthcare centres (PHCs). Two groups, namely, ordinary collaborative stepped care (oCSC, *n* = 456) and *mobile message* and reminder + collaborative stepped care (mCSC, *n* = 439) in the management of major depression in the PHCs.	The mCSC group had significantly better adherence rate when compared with the oCSC group at 6 months (90.0% vs. 67.8%, ARR: 1.31, 95% CI: 1.22–1.40) and at 12th month (78.1% vs. 59.2%, ARR: 1.30, 95% CI: 1.20–1.43). Regarding the secondary outcomes, the mCSC group had significantly higher recovery rate, quality of life and retention in treatment and was more cost-effective when compared with the oCSC. The clients were also satisfied with services given via mobile phones.	Intervention	Asynchronous (e.g. text messages)
5.	Gureje et al., 2019	This was a cluster randomised controlled trial to compare high-intensity treatment (HIT) and low-intensity treatment (LIT) on perinatal depression in primary care setting. The unit of randomisation was the primary maternal care clinics while the unit of analysis was the participating pregnant women. The LIT consisted of (1) usual care and (2) Mental Health Gap-Intervention Guide (both were referred to as enhanced usual care). The HIT consisted of (1) enhanced care, (2) stepped care and (3) *use of mobile phone voice messages* for follow-up and to remind participants in the arm about home works. Main outcome was changes in the Edinburgh Postnatal Depression Scale (EPDS). About 686 participants (452 = HIT and 234 = LIT).	About 85% completed the follow-up. Participants in the HIT arm higher proportion of remission when compared with the LIT arm, 70% versus 66%, risk difference, 4% (95%, 4.1%, 12.0%), AOR 1.12 (95% CI: 0.73–1.72). HIT was more effective for severe depression (OR: 2.29; 95% CI: 1.01–5.20, *p* = 0.047) and resulted in a higher rate of exclusive breastfeeding of the infant.	Intervention	Asynchronous (e.g. voice messages)
6.	Thomas et al., 2017	The study measured the effectiveness of *short message services (SMS)* reminders on missed appointments in mental health setting. It was a randomised controlled trial involving 200 persons with first-episode psychosis (100 persons per arm). Two groups (intervention arm: appointment in the card + SMS; control arm: card appointment alone). Analysis was per protocol. One hundred and ninety-two persons completed the study.	After adjusting for sociodemographic and clinical variables, SMS reminder independently reduced the risk of missed appointment by 50%.	Intervention	Asynchronous (e.g. text messages)
7.	Oguoma et al., 2020	The study developed an *algorithm* for the diagnosis and treatment of mental health conditions in Nigeria. The authors used structural system analysis design (SSAD) to observe three tertiary hospitals in Nigeria. Data were analysed using computer software. An expert system for diagnosis and treatment using Hypertext PreProcessor (PHP), Hypertext Markup Language (HTML), Cascading Style Sheet (CSS) and Ajax Programing languages while backened was carried out with MySQL.	The authors developed a user-friendly algorithm for online diagnosis and treatment for mental disorders.	Intervention	Asynchronous (e.g. using computer-based algorithm)
8.	Ojagbemi et al., 2022	This was a qualitative study aimed at exploring the views of primary healthcare workers on the feasibility, acceptability and benefits of *smartphone-based* clinical guidance and the e-Mental Health Gap Action Program-Intervention Guide (e-mhGAP-IG) in the management of people with mental health conditions in Nigeria. About 12 primary health centres (both rural and urban) in the South-Western Nigeria participated. About 34 in-depth key informant interviews with two focused group discussions involving a wide range of primary healthcare practitioners. Thematic analysis was used.	Most participants reported that the apps were deployed for purposes other than clinical consultation and decision-making. They also noted that app-based decision-making tool was preferred to the article by the primary healthcare workers. They suggested that future usage of the e-mhGAP-IG would be facilitated by training and supporting of staff, helpful design features and obtaining patients’ buy-in.	Implementation	Synchronous (i.e. telephone conversation)
9.	Kola et al., 2022	This was a qualitative study to design a theory-informed *mobile phone* supported intervention for adolescents with perinatal depression. It utilised focused group discussion and implementation strategies to examine the barriers and facilitators. Analysis was by the theoretical domain framework and the capability, opportunity, motivation and behaviour model.	The participants highlighted the usefulness of the weekly mobile phone calls to support the interventions given.	Implementation	Synchronous (i.e. use of phone calls)
10.	Buhari et al., 2021	The study assessed the willingness and preference for *internet-based* mental health interventions among university students. Two questionnaires, the Mental Health Literacy Questionnaire (MHLq) and the General Health Questionnaire (GHQ-12) were completed by 3300 undergraduate students. A total of 3179 analysable questionnaires.	Preference for internet-based mental health intervention compared to face-to-face intervention was 27.7%. A total of 48.6% respondents were willing to use internet-based mental intervention. Predictors of willingness to use internet-based support: monogamous family setting (aOR = 1.486), help-seeking behaviour (aOR = 2.683), probable mental illness (aOR = 0.333). Predictors of preference for internet-based intervention over face-to-face therapy were younger age (aOR = 1.377), female gender (aOR = 1.342), satisfactory relationship with mother and father (aOR = 1.607 and 1.466 respectively), self-help strategies (aOR = 0.713) and good knowledge of mental health (aOR = 1.610).	Implementation	Synchronous (e.g. video conferencing)
11.	Nwaogu et al., 2021	The study assessed the barriers and motivators for using *digital mental health interventions* among construction workers. It was a qualitative design involving 62 construction workers selected using convenience sampling. Inductive content analysis was used.	Two broad barriers to use of digital interventions were reported, namely, adoption barrier (e.g. lack of awareness and knowledge) and barriers to persistent use (e.g. usability, efficiency and effectiveness, high cost, and boring due to lack of contact). Motivators were broadly into two categories, namely, intrinsic (usability, efficiency and effectiveness) and extrinsic. Participants opined that including features such as comedy skits, music, news, educational and motivational posts in the mental health apps will improve its use.	Implementation	Synchronous and asynchronous
12.	Kola et al., 2021	The study measured the willingness of perinatal adolescent girls to use *digital mental health services*. It was a cross-sectional survey of 260 adolescent mothers.	Majority of the participants responded as being ‘interested’ and ‘very interested’ in the use of mobile phones for preventive interventions (96.2%) and treatment (93.5%) information on mental illness such as depression. Slightly above half preferred to receive such information via text messages.	Implementation	Synchronous and asynchronous
13.	Adeniji et al., 2022	This study aimed to implement the use of *machine learning* to predict the possibility of developing mental disorders. Two models were used, namely, hybrid random forest (HRF) and artificial neural network (ANN) to predict mental illness among information technology staff using data collected.	The random forest (RF) and the hybrid (RF + ANN) models predicted the possibility of an IT staff developing a mental disorder to be 84.5% (for precision, recall and accuracy) and 82.5%, respectively.	Implementation	Asynchronous (computer-based app)
14.	Ogunseye et al., 2022	This study aimed to determine the validity of *machine learning* models to predict treatment outcomes in mental disorders. Several models were used, including bagging, stacking, K-nearest neighbour, Tree class, random forest and AdaBoost.	AdaBoost has the highest predicting accuracy (81.75%) for treatment outcomes in mental illness.	Implementation	Asynchronous (computer-based app)

CI, confidence interval; s.d., standard deviation; ARR, absolute risk reduction; aOR, adjusted odds ratio.

## Main findings and thematic areas

Majority (77.8%) of publications were conducted in the South West region of the study country, while the South East and South South regions had one publication each originating from those zones. There were no studies identified to have been conducted in any of the northern regions of the country. All, with the exception of one (internet-based), were studies utilising phone calls or SMS. Two themes were evident, namely, implementation and interventions. These areas of focus are summarised in the following sections.

### Theme 1: Intervention studies on the use of digital psychiatry in mental healthcare services in Nigeria

A total of seven studies (*n* = 7) were found in this area. Most of these studies used phone calls and text messages in addition to other interventions to improve perinatal depression^11,12,18^ and major depressive disorders in student populations. These studies consistently reported significant improvement in depression symptom ratings in the arm that involved the use of phone calls and text messages. One study examined specifically the effectiveness of text message reminders on first visit clinic attendance for persons with first-episode psychosis.^13^ The authors found that after adjusting for potential confounding variables, use of text message reminders reduced missed first appointment visits by 50%.^13^ The other two articles reported on the use of computer-based algorithm in making diagnosis of major mental disorders^19^ and reasons for distress calls in a suicide prevention helpline while delivering counselling and linkage services using dedicated mobile helpline,^20^ respectively.

### Theme 2: Implementation studies on the use digital psychiatry in mental healthcare services in Nigeria

Seven studies (*n* = 7) focused on various implementation outcomes, namely, acceptability and feasibility of the use of digital platforms for mental healthcare services in Nigeria. A good number of these studies (*n* = 4) reported on the acceptability and preference of the use of digital technology for treatment by the patients,^21,22,23,24^ while one study (*n* = 1) reported feasibility and acceptability of same digital resource among primary healthcare workers.^25^ Two studies (*n* = 2) reported on the feasibility of using machine learning models to predict mental disorders and treatment outcomes.^26,27^

## Discussion

The major takeaways from this scoping review are twofold: firstly, intervention studies consistently noted the advantages of using digital technologies to offer care, reduce mental health symptoms and increase clinic attendance. Secondly, implementation studies demonstrate that despite certain difficulties encountered, the use of digital technologies in Nigeria to make diagnoses, increase clinic attendance and provide mental health treatments is acceptable and practicable.

The finding that digital mental healthcare services are useful is in line with global studies.^21,22^ Digital technologies have been demonstrated to be efficient in diagnosis and assessment across a range of populations, enhancing clinic attendance and delivering treatments^21,22^ in various reviews encompassing studies conducted all over the world. According to several researches, it is both similar to in-person care and even more highly favoured by patients.^28,29^ The coronavirus disease 2019 (COVID-19) pandemic paved the way for greater use of digital mental health interventions and innovations as part of routine care offered by therapists.^30,31,32^ However, the impact of digital mental health interventions on therapeutic alliance, a concept triad by Bordin that consists of therapist-client bond, agreement on goal-directed tasks and agreement on therapeutic goals,^33^ has been debated. While there are concerns that digital mental health interventions may erode the value of therapeutic alliance cultivated by the traditional face-to-face therapies, it is suggested that therapeutic alliance can also be cultivated in the digital context, leading to an increased engagement and adherence to digital interventions.^33,34,35^

The results of this review are consistent with earlier studies showing that telehealth, including telephone-based services, is feasible and acceptable for patients with severe mental illness and may be an important teaching tool for mental health professionals.^29,30^ These findings are also relevant as to whether digital psychiatry is acceptable and feasible in Nigeria.^36,37^ The COVID-19 pandemic, access to mobile and smartphones, and other variables, such as the viability and acceptance of digital technology in the delivery of mental healthcare, as highlighted in this research, are likely fuelling the expansion of mental health tech startups like PsyndUp in Nigeria.

The findings of this review have implications for mental healthcare services in Nigeria. For example, utilising the opportunities provided by the usage of digital psychiatry may help close the treatment gap for mental, neurological and substance use disorders, which still surpasses 80%.^38^ Thankfully, the time seems right because there is a noticeable penetration of telecommunications services in Nigeria.^10^ According to recent statistics, Ghana, Nigeria, South Africa and Kenya are among the African nations with telecommunication densities that are above 100%. Parallel to this, it is anticipated that smartphone connections in SSA will nearly double in the same period, from 816 million in 2019 to 1.05 billion in 2025.^10^ To close the treatment gaps in Nigeria, these infrastructures could be used to scale up digital mental health services. With the help of these infrastructures, digital mental health services could be expanded throughout Nigeria, helping to bridge the treatment gap. Notwithstanding the excitement, there are difficulties in Nigerian digital psychiatry practice.

Concerns about client privacy and rights when providing mental healthcare services online are a major problem. Nigeria currently has no defined policies, protocols or procedures in place. The Nigeria Data Protection Regulation (NDPR) was released in 2019 by the National Information Technology Development Agency (NITDA), a federal organisation tasked with overseeing electronic governance and monitoring in Nigeria.^39^ The preservation of natural persons’ rights to data privacy is one of this guideline’s main goals. The NDPR’s data protection framework, on the other hand, does not particularly offer security for personal health data or for the complete cycle of data collection, processing, retention and deletion. This is crucial, particularly in healthcare settings given the distinctive characteristics of medical data. To protect doctors and patients and improve the delivery of digital mental healthcare, this flaw in Nigeria’s legal framework needs to be fixed. The sustainability of funding for digital health is a significant issue. Sustainable finance has been cited to be a significant barrier to the expansion of telemedicine in Africa.^40^ Nonetheless, suggestions have been made for alternate funding sources that may be used for sustainability such as healthcare impact bonds, diaspora bonds and charity bonds.^40^ In order to promote partnerships with the commercial sector and the diaspora and facilitate telemedical services in Nigeria, significant political will is additionally required.

Based on the aforementioned, the authors recommend using digital technologies to enhance mental healthcare services for all age groups. In addition, a legislative framework must be established in Nigeria to take advantage of the media’s potential for improving the provision of mental healthcare there in the face of a severe dearth of mental health professionals. Also, more study is required to evaluate various models for combining digital tools with Nigeria’s standard face-to-face delivery of healthcare.

### Limitations

While the scoping review methodology provided a comprehensive overview of the existing literature on digital psychiatry in Nigeria, some limitations must be acknowledged. The absence of a rigorous quality assessment of the included studies may impact the overall reliability of study findings. The scoping review design prioritises breadth over depth, thus not allowing for an exhaustive analysis of the methodologies, results or limitations of individual studies. The scoping review is a snapshot of the literature at a specific point in time. Given the rapidly evolving nature of digital psychiatry interventions and research, some newer studies or emerging trends may not be adequately captured in our review.

## Conclusion

Digital technology seems acceptable to Nigerian patients and clinicians and its approaches have been successfully used in the treatment of common mental health conditions and to improve clinic attendance. An important step in facilitating its use in routine care of Nigerians with mental health disorders would be to first engage in research to address several notable implementation barriers. More studies are needed to identify regional gaps, and the quality of evidence from the bulk of studies needs to be improved upon.
